# Attachment discs of the diving bell spider *Argyroneta aquatica*

**DOI:** 10.1038/s42003-023-05575-7

**Published:** 2023-12-06

**Authors:** Clemens F. Schaber, Ingo Grawe, Stanislav N. Gorb

**Affiliations:** Functional Morphology and Biomechanics, Zoological Institute, Kiel University, Am Botanischen Garten 9, 24118 Kiel, Germany

**Keywords:** Biomechanics, Animal behaviour

## Abstract

To adhere their silk threads for the construction of webs and to fix the dragline, spiders produce attachment discs of piriform silk. Uniquely, the aquatic spider *Argyroneta aquatica* spends its entire life cycle underwater. Therefore, it has to glue its attachment discs to substrates underwater. Here we show that *Argyroneta aquatica* applies its thread anchors within an air layer around the spinnerets maintained by superhydrophobic setae. During spinning, symmetric movements of the spinnerets ensure retaining air in the contact area. The flat structure of the attachment discs is thought to facilitate fast curing of the piriform adhesive cement and improves the resistance against drag forces. Pull-off tests on draglines connected with attachment discs on different hydrophilic substrates point to dragline rupture as the failure mode. The Young´s modulus of the dragline (8.3 GPa) is within the range as in terrestrial spiders. The shown structural and behavioral adaptations can be the model for new artificial underwater gluing devices.

## Abstract (web summary)

Multiple behavioral and functional adaptations enable the unique water living spider *Argyroneta aquatica* to apply its silk attachment discs for anchoring its dragline within an air layer around its spinnerets under water.

## Introduction

Silk has enabled the spiders to spread to almost every terrestrial ecosystem on Earth^1^. Maybe the most important factor for the successful use of silk by spiders has been the co-evolution of the silk material properties and the improvement of the silken anchorages, the so-called attachment discs, which are used to glue the silk within the web in orb web spiders and to other materials in the majority of spiders^2–4^. The attachment discs are applied by the spiders mainly to prevent falling during locomotion by fixing the dragline onto the substrate and within the web and for anchoring web constructions^5,6^.

Attachment discs consist of fibrillary silk and a hydrocarbon-rich cement coating^7–9^. These two components are secreted by the piriform glands, released by the piriform spigots on the anterior spinnerets, and applied to the substrate with species specific spinneret movement patterns^10–14^. While walking and climbing, attachment discs are usually applied quite often and need to stick rapidly and reliably to a broad variety of surfaces with different chemistry and topography^15^. The adhesive performance of the attachment discs provides high safety factors on substrates within a large range of surface polarity^10,16,17^. For the attachment discs, previous studies stated a unique combination of optimized principles in the field of lightweight construction such as a shifted point of load application, multiple peeling, thin film peeling, and nanofiber loading in the attached cementation^3,12,18–20^.

**Fig. 1:**
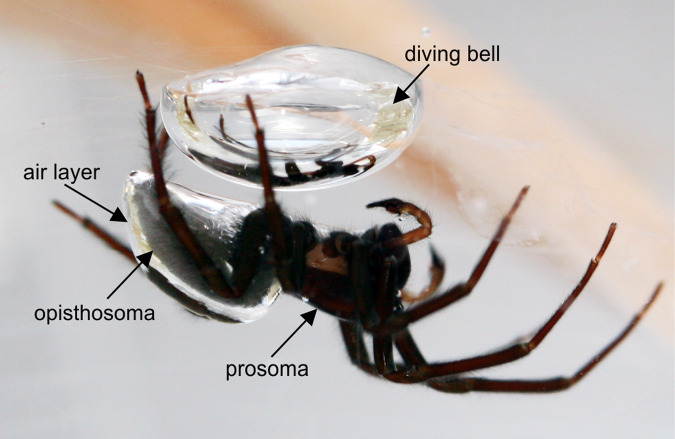
*Argyroneta aquatica* during building of a diving bell underwater. Air trapped by the hydrophobic hair coat of the opisthosoma and ventrally of the prosoma is transported from the water surface to the diving bell.

All of the knowledge about attachment discs so far has been gained from terrestrial spiders. Not much is known about the attachment discs of the water spider *Argyroneta aquatica* (Clerck, 1757)^21^, which is the only spider species that spends its full life cycle underwater. As its housing underwater, *Argyroneta* builds a dome shaped silk web, the so-called diving bell, which is used for resting, mating, and as shelter for cocoons and hatchlings^22–25^. During its construction, the diving bell is actively filled with air transported from the water surface exploiting the non-wettability of hydrophobic hair on the spider´s body^24,26^ (Fig. 1). *Argyroneta aquatica* constantly builds a network of silk threads that are used as dragline, as guidance ropes for locomotion in the home range, as signal transmitters for prey capture, and as the frame for the construction of the diving bell^27,28^. To be able to fix the silk to plants and other objects underwater, *Argyroneta aquatica* needs a special strategy to weave its attachment discs and attach them to the substrates.

The aim of this study was to examine the mechanisms that enable the water spider to produce reliable attachment discs underwater. It was found that *Argyroneta aquatica* applies its silken thread anchors in contact with the substrate within a trapped air bubble maintained around the spinnerets by an arrangement of superhydrophobic setae. Simultaneous symmetric movements of the spinnerets ensure retaining the air in the substrate contact area during the spinning process. Specific morphological adaptations of the attachment discs to their underwater use are their flat structure, which may enable fast curing of the piriform adhesive cement and provide resistance to the drag forces that are stronger in water than in air. The interweaving of single dragline fibers with the piriform silk is thought to provide tight adhesion of the flat structure. Furthermore, the performance of the dragline and of attachment discs spun on glass underwater and outside of the water and on more hydrophobic polystyrene underwater was determined by measuring their maximum breaking force. No statistical differences were found for the force of failure, which points to the suitability of *Argyroneta aquatica*´s attachment discs to stick to many surfaces such as wood and plants underwater. Finally, the elastic modulus of the dragline has been estimated and shown to be of the same magnitude as in terrestrial spider species.

## Results

### Weaving of the attachment discs

**Fig. 2:**
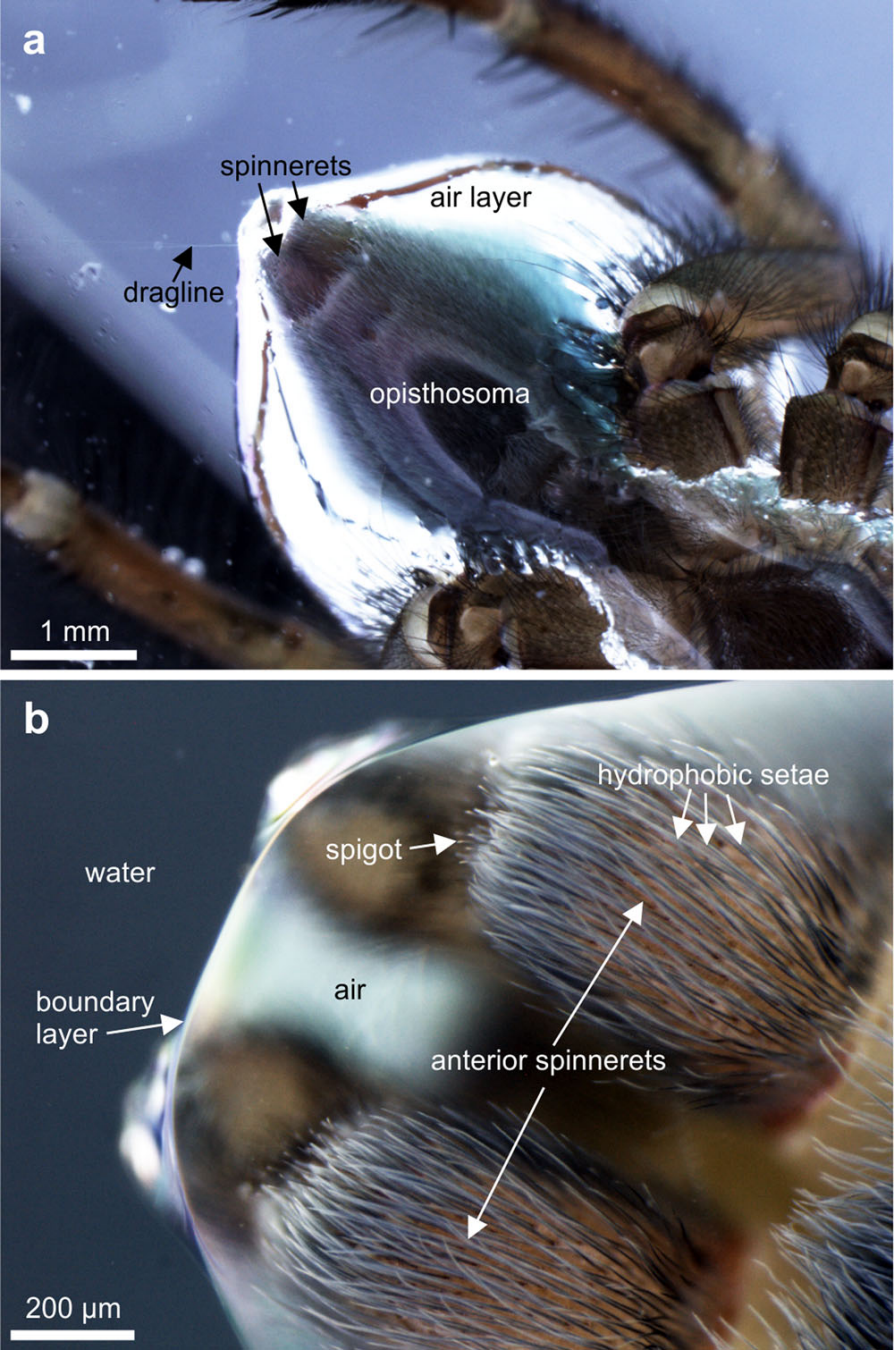
Ventral view of the opisthosoma and the spinnerets of *Argyroneta aquatica* covered by the air layer underwater. **a** Reflected light photomicrograph of the opisthosoma and the shiny white air layer around it and the base of the leg coxae. The distal leg parts are not surrounded by air. **b** The anterior spinnerets including the spigots for spinning of the attachment discs are inside of the air bubble close to the boundary layer with the surrounding water.

Underwater, the dense coat of hydrophobic setae on the opisthosoma and the base of the legs keeps the boundary between water and air at a distance from the cuticle surface of the aforementioned body parts (Fig. 2a). The spigots on the distal ends of the anterior spinnerets are surrounded by hydrophobic setae and do not get in contact with water, too (Fig. 2b).

**Fig. 3:**
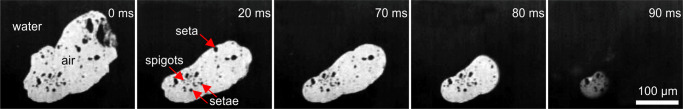
Stills of a high-speed video recording of the air layer in contact with a polystyrene surface under coaxial illumination. From left to right the spigots of the anterior spinnerets, the surrounding hydrophobic setae, and the air layer are pulled off the surface by lifting the opisthosoma at the end of weaving the attachment disc.

Microscopic observations of the attachment site during weaving of the attachment discs through translucent substrate show contact of the spigots of the anterior spinnerets and the surrounding hydrophobic setae inside of the air layer (Fig. 3). Movements of the spinnerets during weaving lead to corresponding shifts of the air-substrate interface.

**Fig. 4:**
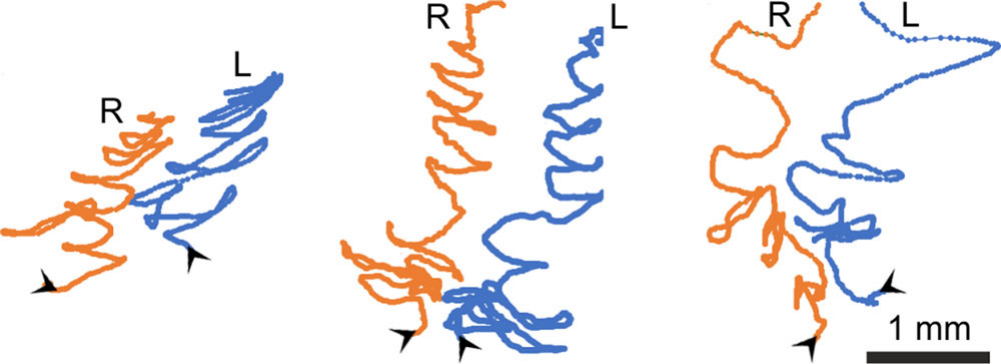
Tracing of anterior spinneret movements from high-speed video recordings of the weaving of three different attachment discs. The orange tracks (R) follow the movement of the tip of the right anterior spinnerets, the blue tracks (L) those of the left ones. The arrowheads mark the starting points and the initial directions of the movements. The time taken for weaving of the individual attachment discs were 1.033 s (left), 1.945 s (middle), and 0.911 s (right).

The weaving of an attachment disc starts with making contact of the spigots with the substrate. For this purpose, the left and the right anterior spinnerets converge and lower onto the substrate. Then the two spinnerets synchronously move away laterally from the opisthosomal centerline and back again to the center continuously, and the piriform silk is spread onto the substrate. Simultaneously, the spider moves its opisthosoma in parallel with the body long axis so that the adhesive piriform substances are distributed over a larger area. The air layer is always kept in between and around the moving spinnerets (Supplementary Movie 1). Finally, the spinnerets are lifted off the substrate and only the dragline extends from its fixation point on the substrate. The number of lateral strokes of the spinnerets varied with a maximum number of nine strokes. Exemplary tracks of spinneret movements during the production of attachment discs are shown in Fig. 4 (source data in Supplementary Data 1) and Supplementary Movie 2. In average, for the production of an attachment disc the spigots moved by 10.8 ± 2.9 mm at a speed of 7.6 ± 4.5 mm s^−1^ (*n* = 12).

In dry conditions outside of the water the movement patterns were the same. However, the spinnerets produced a small ravel of silk already before the spigots were lowered down onto the substrate during the so-called pre-movements as reported earlier for other spider species^12,16,22^. This small amount of silk was then attached to the surface at the beginning of the production of fresh piriform silk for weaving of the new attachment disc.

### Structure of the attachment discs

**Fig. 5:**
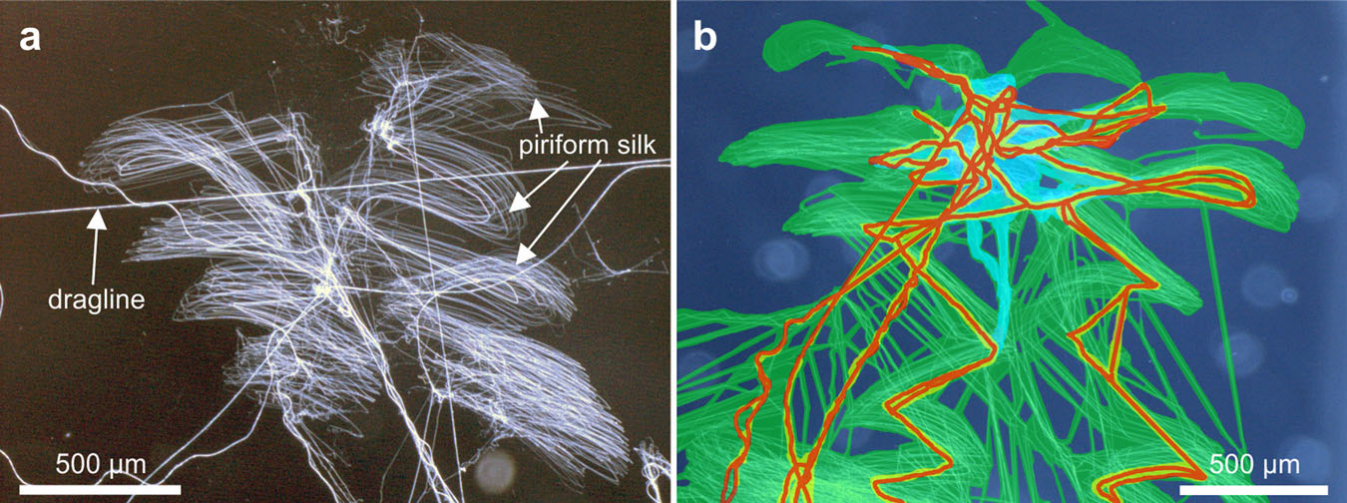
Attachment discs of *Argyroneta aquatica*. **a** Light microscopic image of an attachment disc on a glass surface. **b** Colored photomicrograph of an attachment disc. The dragline (red) is attached to the glass surface by a meshwork of adhesive piriform silk (green). The yellow outlines of the dragline silk strands indicate contact with piriform secretions. The light bluish region in the upper center of the image marks silk without surface contact as determined using the focus depth of the microscope during examination.

Figure 5a shows a typical attachment disc of *Argyroneta aquatica*. The mostly symmetric lateral movements of the spinnerets lead to a symmetric pattern of piriform silk. The attachment discs of the water spider are rather flat and do not show a pronounced elevated bridge structure that connects the piriform silk with the dragline. Such bridge structures are suggested to be an adaptation in terrestrial aerial web building spiders by increasing the attachment discs´ resistance to tensile loads of the dragline in varying angles^13^. Single fibers of dragline silk are continuously interweaved with the piriform silk during the application of the attachment disc, and their courses follow the movements of the anterior spinnerets (Fig. 5b).

**Fig. 6:**
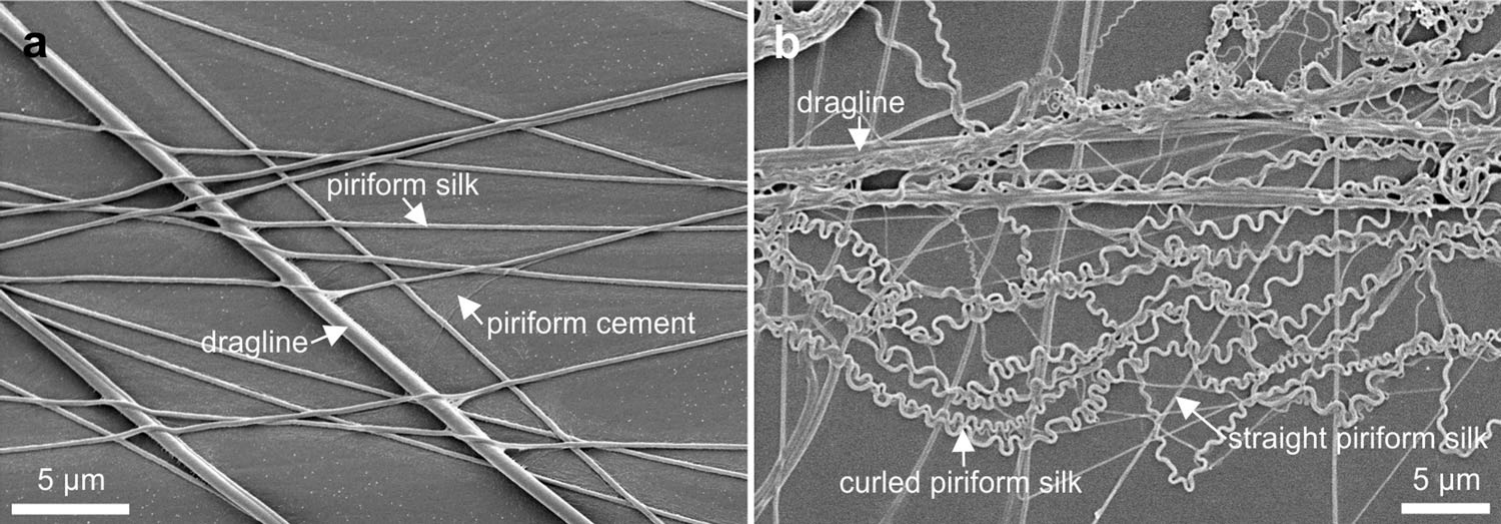
Scanning electron micrographs of attachment disc silk of *Argyroneta aquatica*. **a** The dragline fibers are taped down by the piriform silk threads, which themselves are glued to the surface by the amorphous piriform cement. The density of piriform silk fibers is low compared to attachment discs of other spider species. **b** A mix of curled and straight piriform silk threads crossing the dragline of an attachment disc spun underwater.

The attached piriform silk fibers are covered with the piriform cement and hold down and fix the dragline to the substrate (Fig. 6a). Additional to the straight piriform silk, many strongly curled piriform silk fibers were found at the outlines of the attachment discs spun underwater (Fig. 6b).

### Strength of the attachment discs and the dragline

Pulling tests were performed in four different testing regimes. In the regimes “wet” the attachment discs were produced by the spider underwater either on glass or on polystyrene to test for possible differences between the two substrates with different surface energy. These measurements were performed with the attachment discs submerged 2 cm in water and the loose end of the approximately 4 cm long dragline fixed to the force sensor outside of the water. In the regime “dry glass” the attachment discs were made by the spider in air without any fluid water contact. The measurements were performed in air outside of the water. To test for the tensile properties of the dragline only, sections of the dragline produced outside of the water were tested in dry conditions in the testing regime “dry dragline”. It was not possible to harvest sections of dragline from silk produced underwater.

**Fig. 7:**
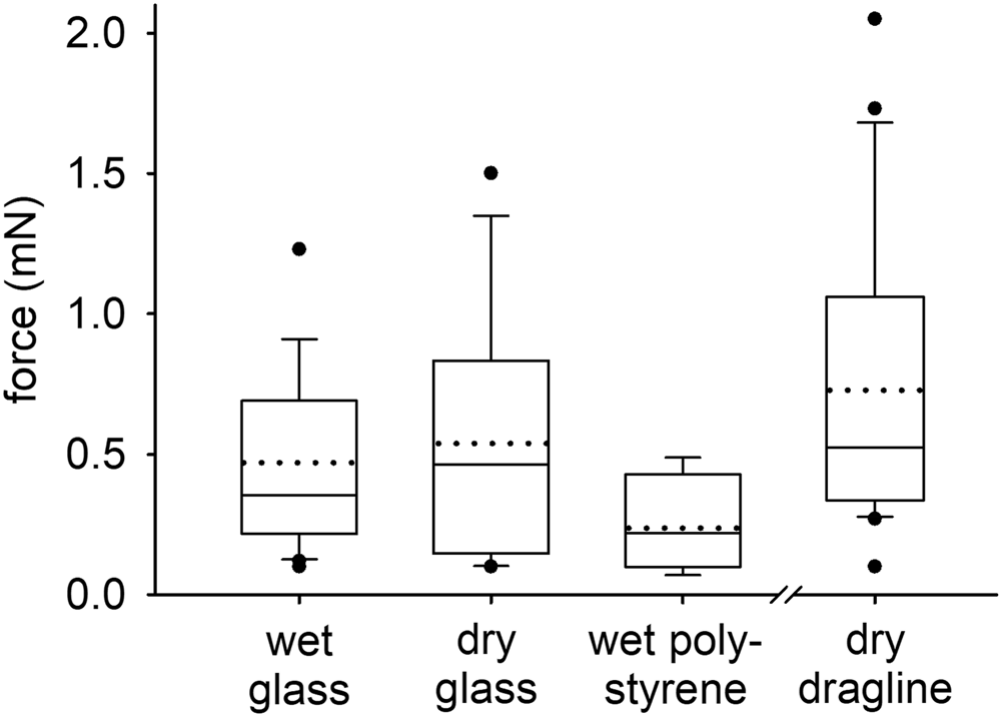
Maximum force before failure of attachment discs and the dragline in tensile tests. The examined attachment discs were weaved on glass underwater (wet glass) or in air (dry glass) or on polystyrene underwater (wet polystyrene). The tensile tests of the dragline were performed in air. The boxes indicate the 25th and 75th percentiles of the data, the whiskers the 10th and 90th ones. The full horizontal lines inside the boxes show the median and the dotted lines the mean values. The single dots are outliers. The data of the pulling tests were collected from six spiders (*N* = 6) and were performed on *n* = 20 (wet glass), *n* = 12 (dry glass), and *n* = 7 (wet polystyrene) attachment discs. The data of the dragline tensile tests are from three spiders (*N* = 3) and *n* = 22 sections of dragline.

For attachment discs spun underwater on glass, the maximum force in the tensile tests before failure was 0.47 ± 0.31 mN (mean ± standard deviation; *n* = 20) with a minimum value of 0.10 mN and a maximum of 1.23 mN. For attachment discs spun underwater on polystyrene the mean value was 0.24 ± 0.15 mN (*n* = 7) with minimum and maximum values of 0.07 mN and 0.49 mN, and for attachment discs spun on glass outside of the water 0.54 ± 0.41 mN (*n* = 12) with a minimum force of 0.10 mN and a maximum force of 1.50 mN. The dragline alone resisted a mean tensile force of 0.73 ± 0.53 mN (*n* = 22) with minimum and maximum values of 0.10 mN and 2.05 mN (Fig. 7; source data in Supplementary Data 2). The statistical comparison of the results of the different tensile test regimes yielded no significant difference neither between the attachment discs spun under different conditions (Kruskal-Wallis one way analysis of variance (ANOVA) on ranks, *P* = 0.148) nor between any of the four groups tested including the dragline (one way repeated measures ANOVA, *P* = 0.118).

There are four single strands of the dragline interweaved within the piriform silk of the attachment disc (Fig. 5b). Therefore, it can be assumed that the dragline consists of these four single fibers bound together in parallel. The thickness of single dragline fibers measured from five different scanning electron micrographs, seven different draglines, and at 35 different straight sections varied between 0.34 µm and 0.72 µm with a mean value of 0.49 ± 0.12 µm. Even within 4 µm of a single dragline fiber its thickness could differ up to 0.08 µm. The mean thickness of the seven measured single dragline fibers varied between 0.37 ± 0.02 µm and 0.69 ± 0.02 µm. When taking the mean value of 0.49 µm of the single fiber diameter and assuming that the fibers of the dragline are circular, the cross sectional area of a typical dragline made up of four single strands was 0.76 µm^2^. Using this mean cross-sectional area and the strongly varying tensile forces for the calculation of the Young´s modulus of the dragline resulted in a mean value of 8.3 ± 5.1 GPa (*N* = 3; *n* = 17) with a minimum of 2.0 GPa, a maximum value of 19.1 GPa, and a median value of 6.9 GPa. The values for the ultimate tensile strength were between 131 MPa and 2.685 GPa with a mean value of 954 ± 690 MPa (*N* = 3; *n* = 22). The mean value of maximum strain of the dragline before breaking was 23.5% ± 3.9% (*N* = 3; *n* = 22) and the minimum and maximum values were 15.3%, and 32.1%, respectively. The source data can be found in Supplementary Data 3.

## Discussion

The most important finding of the present study is that the water spider *Argyroneta aquatica* produces its attachment discs underwater inside of the air layer maintained by the superhydrophobic hair coat around the opisthosoma and the spinnerets. Superhydrophobic properties of the hierarchically structured spider hair have been reported previously^29^. The spigots extruding the adhesive secretions are always kept dry, which underlines the commonly known secondary switch of the habitat of *Argyroneta aquatica* from terrestrial to aquatic. Therefore, compared to terrestrial spider species, no fundamental chemical modifications of the piriform silk of *Argyroneta aquatica* should be necessary for its application, and especially curing, even if the spinning is done underwater. Other animals that apply adhesive secretions underwater, such as the blue mussel *Mytilus edulis* and barnacles also establish a nanoscopically dry space for the application of their adhesive cement underwater, because the solvent for their attachment secretions is water-based and the exposure to water before curing would decrease their strength^30–33^. For underwater locomotion, some terrestrial beetles trap an air bubble between the setae of their adhesive footpads that de-wets the surface of the substrate at contact. It has been shown that these beetles then apply their adhesive footpad secretion from the tips of the setae to the de-wetted surface area^34^. This principle has already been transferred to biomimetics and technically realized in bioinspired adhesive systems capable of adhering underwater^34,35^.

The hair surrounding the spigots on the spinnerets could also be used to brush away loose dirt particles from the substrate surface in order to provide a reliable clean area for the piriform secretions to attach to. A tactile sensory function of the hair around the spigots, as found for a multitude of spider setae^36–38^, for monitoring the surface morphology of the substrate seems likely. Additionally, the numerous individual slit sensilla, surrounding the spigots of the major ampullate glands, may contribute to the sensing of attachment strength of the dragline to the substrate^39^.

A further adaptation to the underwater habitat may be the application of the piriform gland secretions as a relatively thin and rather two-dimensional layer compared to the highly three-dimensional attachment discs of most terrestrial spiders^16^. The reduced curing time of the piriform cement of such a thin layer is an advantage for its application within the air layer maintained around the spinnerets. The setting of the water-based piriform cement, which contains proteins and carbohydrates in terrestrial spiders^7,8,40^, in the air microenvironment is essential, because it would lose its functionality if it was affected by water contact immediately after production. Furthermore, the flat structure of the attachment disc does not offer a large area of attack for drag, which is much higher underwater than in air. This property may be important for maintaining the stability of the attachment discs of *Argyroneta aquatica* for longer time periods. According to previous experimental and numerical results on the robustness of spider silk anchorages^41^, attachment discs structured like that of *Argyroneta aquatica* are not well suited to resist high loads at steep pulling angles. In its habitat, *Argyroneta aquatica* prefers still waters with dense vegetation, where its web structures are protected from high velocity water flow and high drag forces.

When the attachment discs are applied during the symmetric movements of the spinnerets, the air layer around them is steadily maintained. The symmetric movement pattern is different from the alternating spinneret movements usually found in terrestrial spiders^10,12,13,16^, and likely helps to keep the spigots of *Argyroneta aquatica* dry inside of the air layer by exploiting the surface tension of the water in contact with the air funnel established between the spinnerets. During application of the attachment discs, the overall variability of the spinneret movements, which do not follow a strict pattern in terms of stroke number, directionality and total length, allows the spider to adapt the attachment disc to surfaces with different properties and shapes as in the natural environment.

The curly piriform silk fibers frequently found in attachment discs produced underwater are thought to have come in contact with water shortly after their extrusion. Thereby, the exposure of the silk protein mixture to the two different media air and fluid water during polymerization may have caused different curing speeds of different sections. These different curing speeds could lead to inner tension forces within the curing silk and consequently to its bending and the curled structure.

There was no statistical difference between the strength of attachment discs spun on the hydrophilic glass on air and underwater and on the more hydrophobic polystyrene underwater. For the spider in its habitat the reliable adhesion of the attachment discs on substrates with different surface properties, such as wood and plants, is essential for anchoring its webs and the dragline during locomotion. However, the maximum forces before failure of the attachment discs in general were lower on polystyrene, which may be an evidence for better adhesion on hydrophilic surfaces. Because of the delicacy of the dragline threads, they could not be examined after the tests to observe their failure mode. In scanning electron micrographs of the attachment discs after the tests, the point of breakage of the dragline could not be determined and no peeled off piriform silk was observed. Therefore, because the force values of the tensile tests of the dragline alone were in the same range as those of the force tests on the attachment discs, rupture of the dragline seems to be the most likely failure mode of the attachment structures. With a mean value of approximately 0.5 mN before failure, the attachment structures of *Argyroneta aquatica* are much weaker if compared with other spiders, such as the large species *Trichonephila*^42^ *(*formerly *Nephila) senegalensis* (36 mN), *Cupiennius salei* (35 mN), and *Argiope trifasciata* (28 mN), and even the smaller *Thomisus onustus* (7 mN) and *Parasteatoda tepdariorum* (3 mN)^16^. One cause for these differences in the tensile force may be differences in dragline thickness as values of 3.35 µm were reported for the dragline of *Trichonephila*^42^ *(*formerly *Nephila) edulis*^43^ and 3.5 µm for *Argiope argentata*^44^ compared with 0.49 µm found for *Argyroneta aquatica* in the present study, which represents an approximately 13-fold difference in cross sectional area.

Possible causes for the large variation of the elastic modulus of the dragline between 2.0 GPa and 19.1 GPa are the different thickness of the single dragline fibers that may also be the result of differently sized individual animals. Previous authors reported that even in single individuals the strength of the dragline is influenced by the force acting on it during extrusion^45^ and by the degree of starvation of the animal^46^. Also the dragline thickness decreases with the speed of extrusion^43^. And it may be the case that the dragline not always consists of four parallel single fibers. The mechanical parameters known for other, terrestrial spider species draglines range from 6.9 GPa to 22.2 GPa for the elastic modulus, from 1 GPa to 1.5 GPa in ultimate tensile strength, and from 20.5% to 39% maximum strain^44,47–51^. Therefore, with the mean values of 8.3 GPa elastic modulus, 0.95 GPa ultimate tensile strength and 23.5% maximum strain, the mechanical performance of the dragline of *Argyroneta aquatica* is well within the lower range of that found for other spider species.

When immersed in water, the dragline silk of terrestrial spiders was found to contract to about half of its length in air by reorientation of amino acids in its protein backbone^52^. This so-called supercontraction in water strongly affects the mechanical properties and leads to a decrease of the elastic modulus to less than half of that measured in air^53^. In the present study, for *Argyroneta aquatica* neither differences in mechanical properties nor contraction of silk were observed for the attachment discs and the connected dragline sections when submerged in water. Future studies with a focus on contraction properties of *Argyroneta aquatica* silk would be interesting. Regarding the molecular structure of the piriform silk, transcriptomics show that the gene expression in *Argyroneta aquatica* is highly similar to terrestrial and semi-aquatic spider species^54^. To find out more about the functional adaptations of *Argyroneta aquatica* silk that are crucial for its unique use underwater, analyses of the chemical composition and arrangement of the protein molecules will be valuable. Considering biomimetic implications, the morphology of the spinning apparatus of *Argyroneta aquatica* could be the model for novel underwater gluing devices e.g. an extrusion nozzle that is surrounded by water repellent pillars so that the glue could be applied underwater in air on the de-wetted areas of the substrate.

## Methods

### Animals

Living spiders of the species *Argyroneta aquatica* were caught in the artificial stream lake Rosensee in Raisdorf near Kiel, Germany, using a dip net mounted on a 5 m telescopic wand. The spiders were found in accumulations of the waterweed *Elodea nuttallii*. The freshly captured animals were transported to the lab in plastic jars filled with lake water. In the lab, the spiders were kept together with waterweed in water in 60 l and 100 l aquaria at a constant room temperature of 24 °C and a 12:12 h light/dark cycle, illuminated by standard aquarium fluorescent tubes. The ground of the aquaria was covered with three different layers of fine and coarse sand, and coarse stones. Once a week the spiders were fed with larvae of the phantom midge *Chaoborus crystallinus* obtained from a local fishing store. For experiments underwater, individual spiders were transferred to tap water with the following chemistry: pH value 7.74, CaCO_3_ 2.76 mmol l^−1^, conductivity at 25 °C 570 µS cm^−1^, Ca 91.6 mg l^−1^, Mg 10.3 mg l^−1^, Na 15.5 mg l^−1^, K 2.8 mg l^−1^, Cl 19.0 mg l^−1^, HCO_3_ 339.3 mg l^−1^, NO_3_ 1.65 mg l^−1^, NO_2_ 0.025 mg l^−1^, PO_4_ 0.07 mg l^−1^, SO_4_ 10.0 mg l^−1^, SiO_2_ 23.0 mg l^−1^ (source: Stadtwerke Kiel AG, Kiel, Germany). Sex of the spiders was not considered in the study design. After the study, the animals were kept in aquaria until they naturally died. No ethical approval was required according to the legal framework (TierSchVersV) of the Department of Justice of Germany.

### High-speed video recordings

**Fig. 8:**
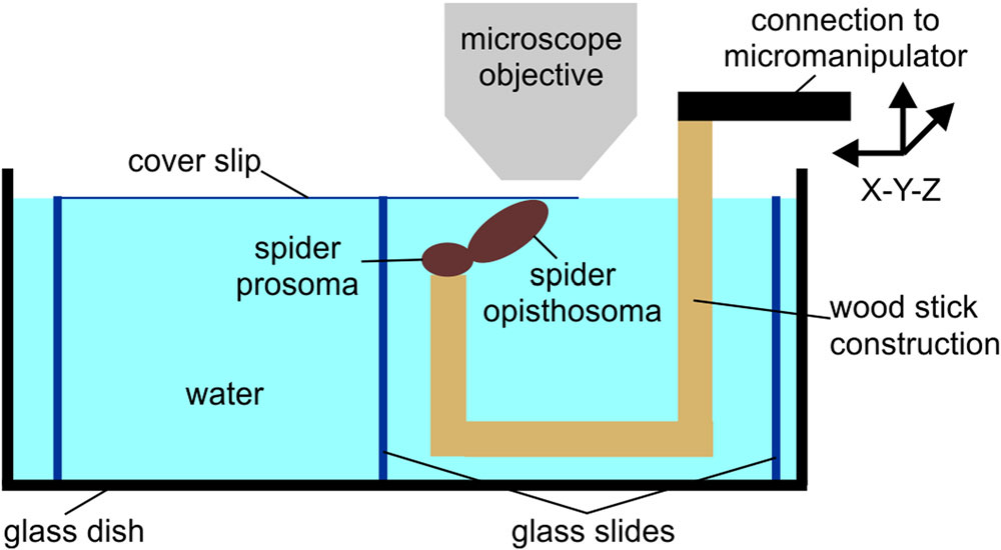
Experimental setup for microscopic video recording of underwater weaving of attachment discs by *Argyroneta aquatica (*schematic side view). The spinnerets on the opisthosoma of the submerged spider are brought in contact with the cover slip in the focus of the microscope using the three-axis micromanipulator. The cover slip is kept horizontally by the construction of glass slides. The glass dish is filled with tap water up to the level of the cover slip.

The weaving of the attachment discs was recorded using high-speed video cameras (Fastcam SA 1.1, model 675K-M1 and Fastcam 1024 PCI, model 100 KC, Photron Europe Ltd., West Wycombe, United Kingdom) mounted either on a stereo microscope (Leica MZ12, Leica Microsystems GmbH, Wetzlar, Germany) or a reflected light microscope (LSM 310, Zeiss AG, Oberkochen, Germany). A cold light source (Leica KL-1500, Leica Microsystems GmbH, Wetzlar, Germany) or two tungsten spotlights (Dedolight CoolH 2x250W, Dedo Weigert Film GmbH, Munich, Germany) were used for illumination. The recording speed of the cameras was set to 500, 1,000 or 2,000 frames per second and the shutter to 1/1,000 s or 1/2,000 s. The spinning of the attachment discs underwater was filmed with the spiders being upside-down like in the natural position. In one set of the experiments the spiders were put freely-movable into a small clear polystyrene flask (Nunclon, Intermed Nunc, Roskilde, Denmark) filled with tap water and filmed from above. In the second set of experiments the spiders were fixed onto a wooden stick using small droplets of a slightly heated mixture of beeswax and colophony dorsally on the prosoma. For precise positioning in the focus of the microscope, the preparation was mounted on a three-axis motorized micromanipulator (DC3001R, controller MS314, World Precision Instruments Inc., Sarasota, FL, U. S. A.). The spiders were immersed inside a water chamber built of microscope glass slides and a cover slip and filmed from above (Fig. 8). Because the spiders did not adhere upside down to glass in air, the weaving of the attachment discs outside of the water was filmed from below with the fixed spiders in upright position standing on their feet, using the same camera setups as described above on an inverted light microscope (Axio Observer.A1, light source HXP 120 C, Zeiss AG, Oberkochen, Germany). The software PFV (Version 3660(x64), Photron Europe Ltd., West Wycombe, United Kingdom) and Photoshop (Version 9.0, Adobe Inc., San José, CA, U. S. A.) were used for recording and editing the video stills. For tracking the motion of the anterior spinnerets in the recorded videos, the plugin MTrackJ (Biomedical Imaging Group Rotterdam, The Netherlands) of the software ImageJ (Wayne Rasband, National Institute of Health, Bethesda, MD, U. S. A.) was used. Further data processing was performed using the software Excel (Microsoft Corporation, Redmont, WA, U. S. A.).

### Microscopy

Photomicrographs were taken using a stereo microscope (Leica M205A, Leica Microsystems GmbH, Wetzlar, Germany) equipped with a digital microscope camera (Leica DFC420). For scanning electron microscopy (SEM), pieces of polystyrene (Petri dish, sterile, Greiner Bio-One International GmbH, Kremsmünster, Austria) with attachment discs on top were air-dried, fixed on SEM sample holders using double-sided carbon-rich adhesive tape and coated with a 10 nm thick layer of gold-palladium using a sputter-coater (EM SCD 500, Leica Microsystems GmbH, Wetzlar, Germany). The samples were viewed using a scanning electron microscope (S-4800, Hitachi Ltd., Tokyo, Japan) at an acceleration voltage of 3 kV. Some images were colored using the software Photoshop (Version 9.0, Adobe Inc., San José, CA, USA).

### Force testing

**Fig. 9:**
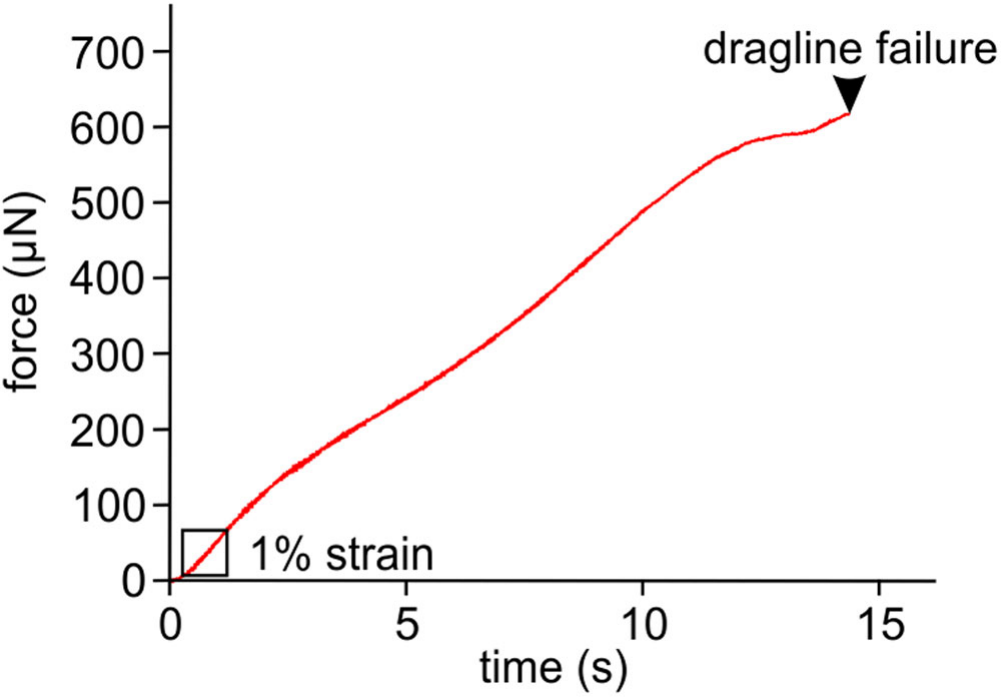
Force-time curve for the determination of the Young´s modulus of the dragline of the diving bell spider *Argyroneta aquatica*. The shown exemplary data are from an initially 58 mm long section of dragline that elongated by 8.9 mm before failure, which corresponds to a maximum strain of 15.3%. For the calculation of the Young´s modulus the force of 1% strain within the initial linear region of the curve (indicated by the rectangle) was used. The stiffness of the dragline in this region was 0.103 N m^−1^. The distance of 1% strain (0.58 mm corresponding to 0.935 s) was calculated using the relationship between the time needed to stretch the fiber by 8.9 mm and the initial length of the section.

Spiders anaesthetized with carbon dioxide were fixed to a wooden stick dorsally on the prosoma using a small droplet of a mixture of beeswax and colophony. Then the spinnerets of the awake spiders were approached to either dry glass or glass or nonpolar polystyrene submerged 2 cm deep in tap water to produce attachment discs on the substrate surface. Subsequently, the spiders were slowly lifted up by approximately 4 cm, and the spun dragline was attached orthogonally to the cantilever of a force sensor (FORT-10, World Precision Instruments Inc., Sarasota, FL, USA) connected to an amplifier and analogue-/digital-converter (Biopac MP-100, Biopac Systems Inc., Goleta, CA, USA). To measure the force needed to break the silk, the force transducer was continuously moved vertically using the motorized micromanipulator at dragline strain rates in the range between 0.0034 s^−1^ and 0.0138 s^−1^. The resulting force curves were recorded to the hard disc of a computer using the software ACQ (Version 3.7.0, Biopac Systems Inc.). Using the same software, the maximum force peak of the curves, shortly before the attachment disc failed, was measured. The tensile performance of the dragline alone was tested in dry conditions on sections harvested in air outside of the water. The procedure of the tensile tests was the same as for the attachment discs, except that the loose end of the dragline was fixed to a laboratory platform using a droplet of beeswax. The relative ambient humidity in the laboratory was 45%. The Young´s modulus *E* of the dragline was calculated using the equation

1 $$E=\frac{F/A}{\Delta L/{L}_{0}}$$

where *F* was the force for extension of the dragline section by 1% of its initial length *L*_0_, *A* the cross sectional area of the dragline fibers as estimated from the thickness measured on SEM images, and Δ*L* the elongation at 1% strain (Fig. 9). The ultimate tensile strength was determined by dividing the maximum force value before failure of the dragline by its estimated cross sectional area. The maximum strain was calculated using the relationship between the distance pulled on the dragline at failure and the initial length *L*_*0*_ of the dragline section.

### Statistics and reproducibility

Statistical analyses were performed using the software SigmaPlot (Systat Software, Inc., San José, CA, USA). Mean values, standard deviations, and median values were calculated using the software Microsoft Excel. The sample size depended on the number and condition of available animals. All tests on the same sample type were repeated seven to 22 times. The samples were from six different animals. The measurements were performed in a pooled random choice. Blinding was not relevant to the study, because the values even within the same sample group varied largely.

### Reporting summary

Further information on research design is available in the Nature Portfolio Reporting Summary linked to this article.

### Supplementary information

Description of additional supplementary files

Supplementary Data 1

Supplementary Data 2

Supplementary Data 3

Supplementary Movie 1

Supplementary Movie 2

Reporting Summary

## Data Availability

All data that support the findings of this study are included in the text, figures, and supplementary material. The source data for Fig. 4 can be found in Supplementary data set 1, the source data for Fig. 7 in Supplementary data set 2, and the source data for the material properties of the dragline in Supplementary data set 3. Further raw and all other data are available from the corresponding author on reasonable request.
