# Supplementary material for: Attachment discs of the diving bell spider Argyroneta aquatica
Source: Commun Biol. 2023 Dec 6;6:1232. doi: 10.1038/s42003-023-05575-7 (PMC10700320; doi:10.1038/s42003-023-05575-7)
Supplement: Supplementary file 1 — Description of additional supplementary files [file 42003_2023_5575_MOESM1_ESM.docx]

Description of Additional Supplementary Files

**File name:** Supplementary Data 1

**Description:** The source data behind figure 4 in the article.

**File name:** Supplementary Data 2

**Description:** The source data behind figure 7 in the article.

**File name:** Supplementary Data 3

**Description:** The source data behind the values for the material properties of the dragline given in the results section of the article.

**File name:** Supplementary Movie 1

**Description:** Opisthosoma and spinnerets of Argyroneta aquatica during spinning of an attachment disc on transparent substrate (33× slow motion).

**File name:** Supplementary Movie 2

**Description:** Tracking of the spinneret motions during the production of an attachment disc in slow motion.
